# Kinetics of Photo-Oxidation of Oxazole and its Substituents by Singlet Oxygen

**DOI:** 10.1038/s41598-020-59889-1

**Published:** 2020-02-28

**Authors:** Nassim Zeinali, Ibukun Oluwoye, Mohammednoor Altarawneh, Bogdan Z. Dlugogorski

**Affiliations:** 1 Discipline of Chemistry and Physics, College of Science, Health, Engineering and Education, Murdoch University, 90 South Street, Murdoch, WA 6150 Australia; 2 Chemical and Petroleum Engineering Department, United Arab Emirates University, Al-Ain, 15551 United Arab Emirates; 3 Office of Deputy Vice Chancellor, Research & Innovation, Charles Darwin University, Ellengowan Drive, NT 0909 Australia

**Keywords:** Chemical engineering, Reaction kinetics and dynamics

## Abstract

Oxazole has critical roles not only in heterocycle (bio)chemistry research, but also as the backbone of many active natural and medicinal species. These diverse and specialised functions can be attributed to the unique physicochemical properties of oxazole. This contribution investigates the reaction of oxazole and its derivatives with singlet oxygen, employing density functional theory DFT-B3LYP calculations. The absence of allylic hydrogen in oxazole eliminates the *ene*-mode addition of singlet oxygen to the aromatic ring. Therefore, the primary reaction pathway constitutes the [4 + 2]-cycloaddition of singlet oxygen to oxazole ring, favouring an energetically accessible corridor of 57 kJ/mol to produce imino-anhydride which is postulated to convert to triamide end-product in subsequent steps. The pseudo-first-order reaction rate for substituted oxazole (e.g., 4-methyl-2,5-diphenyloxazole, 1.14 × 10^6^ M^−1^ s^−1^) appears slightly higher than that of unsubstituted oxazole (0.94 × 10^6^ M^−1^ s^−1^) considering the same initial concentration of the species at 300 K, due to the electronic effect of the functional groups. The global reactivity descriptors have justified the relative influence of the functional groups along with their respective physiochemical properties.

## Introduction

**Figure 1:**
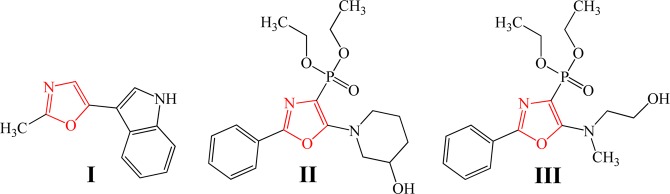
Chemical structures of oxazole derivatives. The red-coloured segments depict oxazole.

Oxazole, an exceptional nitrogen-containing heterocycle, serves as the building blocks of a variety of unique and highly functional natural and artificial products. This five-membered aromatic heterocycle holds implications for extensive research efforts in synthesising highly significant pharmaceuticals^1^ and agrochemicals^2^. Even though parent oxazole does not occur in natural products, its structurally complex substituents transpire in biologically active structures and marine micro-organisms^3^. For instance, as illustrated in Fig. 1, pimprinine (structure I), representing a remarkable antiepileptic compound, is analogous to 2-methyl-5-phenyloxazole in chemical structure. Likewise, phosphorylated derivatives of oxazole (i.e., structures II and III) serve as photosynthetic stimulators, certifying the important application of oxazole moiety in biological activities, germination of plant seeds and growth of vegetative organs^4^. Oxazole-containing amino acids also occur in natural cyclic peptides which are fundamentally sourced from marine organisms^5^.

Generally, oxazole segments represent highly reactive units that efficiently participate in photo-oxidation by singlet molecular oxygen to produce compounds such as isoimides^6^ and nitriles^7^ under mild atmospheric conditions. One of the significant applications of photo-oxidation of oxazole groups involves the synthesis of antimycin A3, a useful biological antitumor, resulted from the reaction of singlet oxygen with the substituted oxazole (2-methyl-4,5-diphenyloxazole)^8,9^. In a broader context, macrocyclic lactones and lactams, with their escalating medicinal values, could mainly be formed upon a simple photo-oxidative rearrangement of oxazole compounds to triamides intermediates^10,11^, accentuating the formidable role of singlet oxygen in procuring vital chemical and natural products^12^.

**Figure 2:**
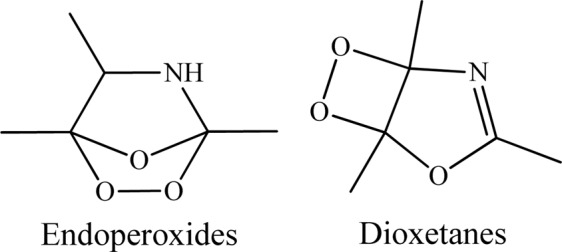
Chemical structures of endoperoxides and dioxetanes.

Oxazole exhibits some unique physicochemical properties exclusively discussed in the Section 3 of this contribution. Therefore, the pronounced chemical reactivity of unsubstituted oxazole towards oxidation by singlet oxygen could be attributed to single electron transfer reactions, as well as many other types of reactions that are not attainable under aerobic conditions at room temperature by the ground state molecular oxygen^13^. Although the addition of singlet oxygen to organic substrates usually follows the *ene*-type, [2 + 2]- and [4 + 2]-cycloaddition reactions, the absence of allylic hydrogens in the molecular structure of oxazole ring should impede the *ene*-type addition of singlet oxygen to such system. Hence, the [2 + 2]-cycloaddition of singlet oxygen to oxazole ring to form of 1,2-dioxetane (Fig. 2) has been excluded as a dominant channel^14^. Accordingly, [4 + 2]-cycloaddition remain the primary reaction route, resulting in the formation of endoperoxides (Fig. 2). A previous study on photo-oxidation of substituted oxazoles by Gollnick and Koegler^15^ endorses the exclusive production of endoperoxides through rapid reactions which are nearly independent of the selected solvents.

This study improves the current state of knowledge pertinent to oxidation of oxazole-derivative compounds by singlet oxygen, employing the density functional theory (DFT) to scrutinise the relative properties and molecular descriptors of unsubstituted and substituted oxazoles. This offers remarkable insights into the mechanistic pathways and kinetic responsiveness of the five-membered aromatic heterocycles to oxidative interactions with the singlet state of dioxygen molecule. The results should help reinforce the biological applications and spontaneous oxidation of oxazole, as well as distinguish the effects of the substituent functional groups.

## Computational Approach

Ab initio quantum chemistry calculation remains an essential method to evaluate the electronic structure and molecular properties of chemical compounds. In this work, the DFT approach efficiently computed the optimised geometry of the molecules and located the transition states through Gaussian 09 package^16^, employing the B3LYP functional in conjunction with the 6–311 ++ g(2df,2p) basis set expanded by d-type and p-type polarisation functions on heavy atoms and hydrogen atoms, respectively, as well as diffuse functions for both hydrogen and heavy atoms. Frequency analysis pinpointed the local minima of the optimised species.

We applied the Yamaguchi^17^ correction technique to eliminate the considerable higher spin-states contamination in singlet diradicals. This procedure appears indispensable to predict realistic energy gaps between singlet-triplet states of open-shell systems. We have shown in our previous study^18^ that the commonly deployed B3LYP functional outperforms meta-hybrid DFT methods in computing singlet-triplet energy gap for a wide range of diradical species.

Material studio DMol^3^ code^19^ afforded the estimation of the frontier orbitals, Hirshfeld^20^ atomic charges and Fukui^21^ (*ƒ*^*−1*^) indices of electrophilic attack. The frontier molecular orbitals theory defines the highest occupied molecular orbital (HOMO) and the lowest unoccupied molecular orbital (LUMO) levels as a measure of molecular excitability from the ground state (*S*_0_) to the relevant excited state (*S*_1_). The reactivity of molecules, in general, inversely correlates with the energy gap between these two frontier orbitals. The gap between HOMO and LUMO energy levels appraised the key features of the system, such as chemical potential (*μ*), global hardness (*η*), global softness (*σ*), electronegativity (*χ*) and electrophilicity index (*ω*). For kinetic considerations, the ChemRate software^22^ applied the master equation solver to compute the activations energies and rate expressions for each mechanistic step over a temperature range of 300–600 K.

## Physicochemical Properties of Oxazole

**Figure 3:**
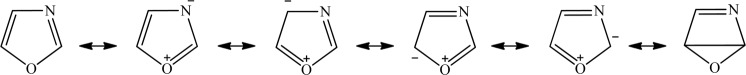
Set of resonance structures of oxazole.

As represented in Fig. 3, oxazole exists in a hybrid resonance state of canonical structures, and hence its aromatic character. The ionic resonance of oxazole structures corresponds to the high reactivity of its ring as well as its considerable tendency to participate in reactions involving both electrophilic and nucleophilic reagents. In fact, oxazole ring cleaves readily to produce acids, amides and imides during oxidation process^23^.

**Figure 4:**
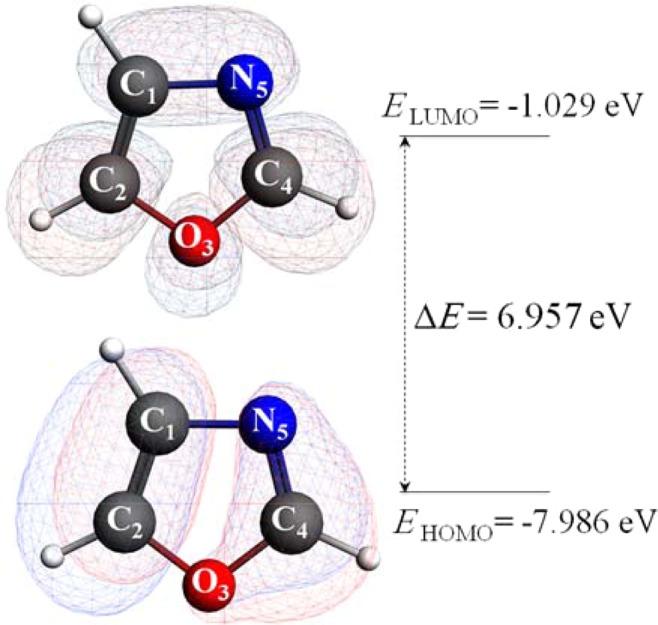
Frontier molecular orbital and energy levels computed in B3LYP/6–311 ++ g(2df,2p) level.

Figure 4 portrays the maps and energy levels of the highest occupied and lowest unoccupied molecular orbitals (i.e., HOMO and LUMO properties) of the core molecule. The HOMO-LUMO gap indicates the chemical reactivity and kinetic stability of a molecular system; so that, the larger the gap, the higher the stability and the lower the reactivity. To put into perspective, the HOMO-LUMO gap of 6.957 reveals that oxazole is more stable (i.e., less reactive) than benzene and furan that retain the energy gaps of 6.720 and 6.572, respectively^24^. From a physical viewpoint, the energy gap between the electronic orbitals signifies the energy required to transfer one electron from the molecular ground state to the excited state, thus, can characterise the chemical and spectroscopic properties of organic molecules such as oxazole^25^.

The electron density in the HOMO is distributed around C_1_ = C_2_ and C_4_ = N_5_ double bonds, while that of the LUMO encircles the C_2_, O_3_ and C_4_ atoms and the C_1_-N_5_ bond. The electrophilic oxidation reaction should initiate via the interaction between HOMO of oxazole and LUMO of singlet oxygen. Thus, HOMO lobes on oxazole distinguish the electron-donor sites (e.g., the C_1_ = C_2_ double bond with the highest electron population) that are relatively most reactive towards singlet oxygen attacks.

**Figure 5:**
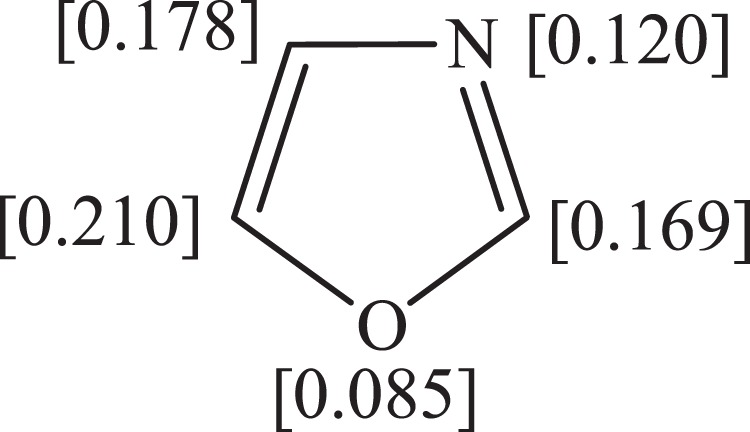
Fukui indices (*f*^−1^) for oxazole.

As shown in Fig. 5, the electrophilic Fukui function indices (*f* ^*−1*^), a credible parameters to locate the most reactive segments of a compound involved in an electrophilic addition reaction, are in accordance with the frontier molecular orbital result, indicating the C_1_ = C_2_ (due to the largest *f* ^*−1*^ value) as the most vulnerable site within the oxazole structure towards an attack by electrophiles.

As earlier stated, the absence of allylic hydrogen in oxazole should eliminate the *ene*-mode addition of singlet oxygen to the aromatic ring. Consequently, the interaction of singlet oxygen with oxazole is expected to exclusively follow the [2 + 2]- and [4 + 2]-cycloaddition processes. These reactions are usually influenced by solvents, substituent functional groups and other reaction conditions^23^. Therefore, the photo-oxidation of oxazole could readily result in cleavage of the aromatic ring, owing to [4 + 2]- and [2 + 2]-cycloaddition of singlet oxygen to the system, which is evidently followed by the formation of triacylamines^7,26,27^.

## Results and Discussion

This section provides the results of DFT calculations performed on both substituted and unsubstituted oxazole to elucidate the molecular electronic characteristics and reaction kinetics of the chemicals in response to singlet oxygen electrophilic attack. The spin operator < *S*^2^ > values for singlet state energy and relevant thermal enthalpies of pure high-spin and broken-symmetry states have been estimated and corrected using the Yamaguchi’s formalism detailed in our previous work^28^.

### Unsubstituted oxazole

#### Geometry and electronic structure

**Figure 6:**
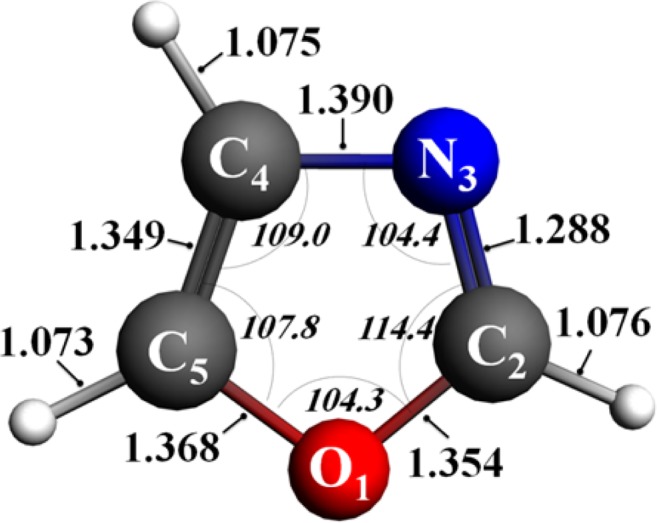
Optimised structure of oxazole derived from DFT B3LYP/6–311 ++ g(2df,2p) level of theory. Bond distances and angles are expressed in Å and degrees, respectively.

Figure 6 depicts the molecular conformation of oxazole as performed at B3LYP/6–311 ++ g(2df,2p) level. These results agree with a previous study conducted by Mukhopadhyay *et al*.^29^ who applied both the (U)B3LYP/cc-pVTZ and (U)M06-2×/cc-pVTZ levels of theory to elucidate the structural aspects of heterocyclic oxazole. As enumerated in Table 1, these values are also in a good accordance with Roche’s *et al*.^30^ result in which an improved linear combination of atomic orbitals (LCAO) method is exploited in theory. The values of 104 degrees for C_2_-N_3_-C_4_ and C_5_-O_1_-C_2_ angles (Table 1) reveal the electronic effects of lone pairs in reducing the angles in the *sp*^2^ hybrid orbitals. However, the carbon-centred angles correlate well with the analogous values for the common five-membered conjugated rings.

**Table 1:** Optimised geometrical parameters of oxazole structure.

Model	(U)B3LYP/cc-pVTZ^29^	LCAO^30^	This work
**Selected bond lengths [Å]**			
O_1_-C_2_	1.355	1.36	1.354
C_2_-N_3_	1.288	1.33	1.288
N_3_-C_4_	1.389	1.39	1.390
C_4_-C_5_	1.349	1.35	1.349
C_5_-O_1_	1.369	1.36	1.368
**Selected bond angles [°]**			
O_1_-C_2_-N_3_	114.6	114	114.4
C_2_-N_3_-C_4_	104.3	104	104.4
N_3_-C_4_-C_5_	109.1	109	109.0
C_4_-C_5_-O_1_	107.9	109	107.8
C_5_-O_1_-C_2_	104.2	104	104.3

The reactivity of the chemicals can also be approximated by the atomic charge density analysis. Therefore, Table 2 contrasts the relative *π*-electron charge densities obtained from various molecular orbital calculations to those acquired in this work. It remains evident that the C_5_ atom embeds the largest electron density, inducing an excess potential for electrophilic substitution at this site. It should be noted that the calculated charge densities are not the definite values of the atomic orbital population, but rather a measure of distribution of charges in the molecular structure.

**Table 2:** π-electron population on oxazole ring atoms.

O_1_	C_2_	N_3_	C_4_	C_5_	Method	Reference
1.660	0.956	1.288	1.005	1.091	SCF-CI	^36^
1.439	1.155	1.169	1.031	1.205	SCF-MO	^37^
1.842	0.982	1.124	0.951	1.101	HMO	^38^
1.694	0.953	1.216	1.048	1.062	DFT-B3LYP	Calculated

#### Mechanistic and kinetic investigation

**Figure 7:**
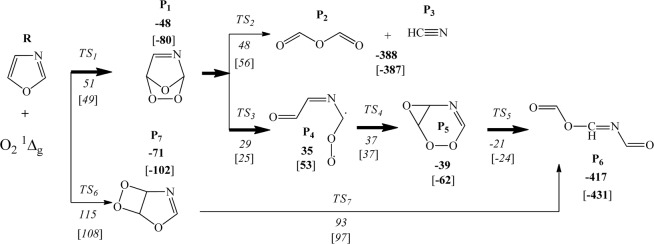
The mechanism for oxazole oxidation by singlet oxygen. Enthalpies are calculated with B3LYP and wB97XD (in brackets) functional at 298.15 K and presented in kJ/mol.

The reaction of singlet oxygen with semi-aromatic oxazole ring commences via [2 + 2]- and [4 + 2]-cycloaddition mechanisms and branches into a variety of channels emerging from decomposition of endoperoxide and dioxetane intermediates. Seemingly, the singlet oxygen *ene*-mode addition to the heterocyclic substrate is inconceivable due to the lack of allylic hydrogen atom and thus, the production of allylic hydroperoxides appears unattainable. Figure 7 collates the proposed mechanism, while subsequent discussion relates to the enthalpic requirements and kinetics of the decomposition and isomerisation channels.

In order to address the effect of dispersion corrections on computed reaction and activation energies, all structures and transition states in Fig. 7 have been reoptimised using the long-range corrected hybrid DFT wB97XD functional^31^ employing the same basis set. Calculated activation energies using the B3LYP and wB97XD methods are relatively close; within 2–7 kJ/mol. This confirms the satisfactory performance of the B3LYP functional in predicting kinetic parameters for the title systems. A noticeable difference in values of reaction energies between the two methods most likely originate from the well-established prediction of slightly expanded structures by standard DFT methods.

The electrophilic [4 + 2]-cycloaddition of singlet oxygen to oxazole ring readily occurs at C_2_ and C_5_ sites, overcoming an enthalpy barrier of 51 kJ/mol, and resulting in the synthesis of unstable bicyclic endoperoxide P_1_. Cleavage of the intermediate P_1_ ring proceeds through either of TS_2_ and TS_3_ pathways, among which TS_3_ embodies slightly lower enthalpy barrier. Thus, the leading ring-opening pathway follows through TS_3_, and triggers the formation of a transient biradical structure P_4_, which subsequently is converted to intermediate P_5_ via a low lying barrier (TS_4_).

**Figure 8:**
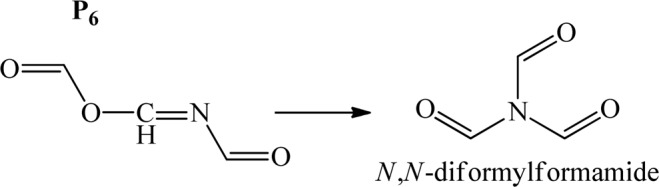
Triamide formation via O–N-acyl migration.

As a whole, the [4 + 2]-cycloaddition of singlet oxygen to oxazole, as the most kinetically favourable step, contributes to the production of imino anhydrides (P_6_), which is further evidenced to undergo an easily accessible intramolecular reorganisation as outlined in Fig. 8, to form reactive triamides (a potent acylating agent)^26^. It has been observed that P_6_ is isolable if oxazole is substituted with functional groups that encompass a saturated ring^32^.

**Figure 9:**
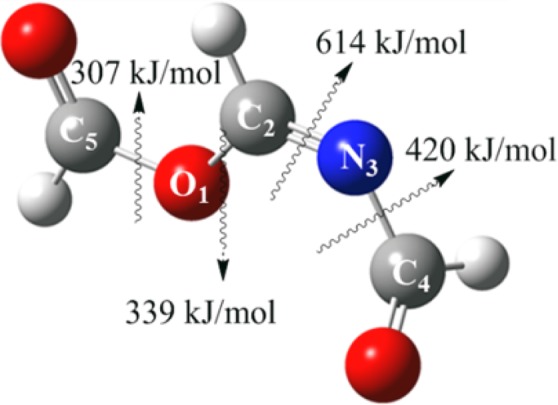
Measured bond dissociation enthalpies for P_6_ structure.

The secondary pathway proceeds through TS_6_, involving the formation of endoperoxide P_7_ via a [2 + 2]-cycloaddition of singlet oxygen to the C_4_=C_5_ bond over an enthalpy barrier of 115 kJ/mol. The simultaneous dissociation of C-C and O-O bonds during a concerted transformation of TS_7_ would mutually give rise to the production of P_6_ intermediate. Furthermore, the bond dissociation enthalpies, as a measure of atomic bond strengths in the polyatomic structure of P_6_ molecule, have been approximated and illustrated in Fig. 9. For this purpose, the thermal enthalpies of the optimised P_6_, as well as the dissociated radical fragments, have been computed by Gaussian 09 code and applied in Eq. 1 to measure the bond dissociation (disruption) enthalpies (*BDE*), where *∆*_*f*_ *H* represents the standard heat of formation (at 298.15 K) and *R* and *X* symbolise the species’ segments breaking into respective radicals.

1 $$BDE(R-X)={\Delta }_{f}H({R}^{\cdot })+{\Delta }_{f}H({X}^{\cdot })-{\Delta }_{f}H(R-X)$$

**Figure 10:**
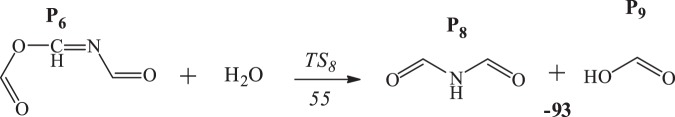
Hydrolysis of P_6_ intermediate (enthalpies are in kJ/mol).

The С_5_-O_1_ bond has the lowest dissociation enthalpy, affirming the fragility of this molecular site. For instance, hydrolysis of P_6_ can readily lead to the formation of N-formylformamide (P_8_) along with formic acid (P_9_) across a moderate enthalpy barrier (TS_8_) of 55 kJ/mol, by direct cleavage of С_5_-O_1_ covalent bond (Fig. 10).

**Figure 11:**
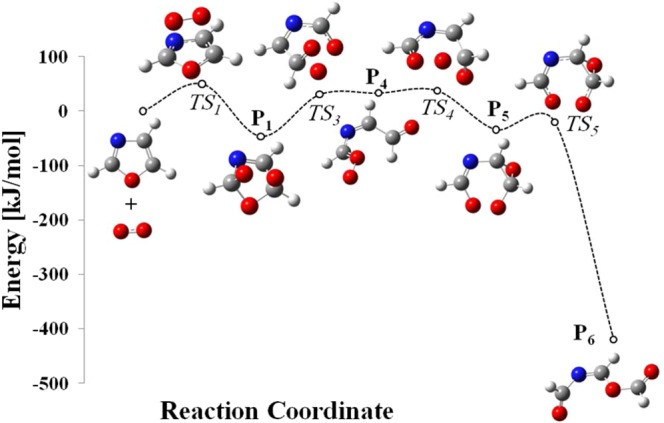
Potential energy surface for oxidation of oxazole as computed at B3LYP/6–311 ++ g(2df,2p) at 298.15 K.

Apparently as shown in Fig. 7, formic anhydride (P_2_) and hydrogen cyanide (P_3_) are the alternative products of the endoperoxide ring cleavage. This process follows a moderate enthalpy barrier (TS_2_) of 48 kJ/mol which slightly overshoots the barrier of major pathway (TS_3_) by 19 kJ/mol. Figure 11 plots the potential energy surface (PES) of the major reaction channel.

Table 3 assembles the reaction rate coefficient for all the unimolecular isomerisation and bimolecular reactions involved in interaction of oxazole with singlet oxygen, fitted to the Arrhenius equation of $k(T)=A{e}^{-{E}_{a}/RT}$ at high pressure limit, within the temperature range of 300 and 600 K (Table 3). The relatively high activation energy of 80 kJ/mol is related to the unimolecular isomerisation of P_1_ → P_4_, which indicates that TS_3_ constitutes the rate determining step.

**Table 3:** Kinetic parameters of oxazole oxidation by O_2_ (^1^Δ_g_).

Reaction	*A* [s^−1^ or cm^3^ molecule^−1^ s^−1^]	*E*_a_ [kJ/mol]
Oxazole + O_2_^1^Δ_g_ → P_1_	1.68 × 10^−13^	58
P_1_ → P_2_ + P_3_	1.01 × 10^14^	100
P_1_ → P_4_	7.99 × 10^13^	80
P_4_ → P_5_	2.44 × 10^11^	4
P_5_ → P_6_	2.17 × 10^13^	21
Oxazole + O_2_^1^Δ_g_ → P_7_	7.60 × 10^−13^	123
P_7_ → P_6_	8.35 × 10^13^	168
P_6_ + H_2_O → P_8_ + P_9_	1.09 × 10^−15^	61

**Figure 12:**
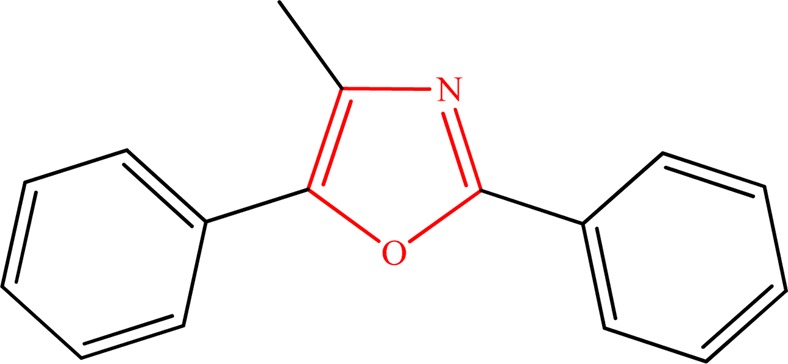
Chemical structure of 2,5-diphenyl-4-methyloxazole.

Wasserman *et al*.^32^ have previously reported the rate constants of 2,5-diphenyl-4-methyloxazole (Fig. 12) photo-oxidation by singlet oxygen in a number of solvents. The proposed *k* values (at 273 K) vary from 2.67 × 10^−14^ cm^3^ molecule^−1^ s^−1^ to 6.51 × 10^−14^ cm^3^ molecule^−1^ s^−1^ from dioxane to benzene solvents, respectively. The corresponding value obtained in this work (Table 3) for the rate determining step, indicates that the rate constant for P_1_ → P_4_ conversion at 300 K equals to 1.56 × 10^−15^ cm^3^ molecule^−1^ s^−1^ following the first order equation of Arrhenius theory:

2 $$k=7.99\times {10}^{13}\exp (\frac{-80000}{8.314\times 300})=0.94\,{s}^{-1}$$

Since P_1_ → P_4_ is a unimolecular reaction, comparison between experimental data and theoretical calculation is applicable through creating a pseudo-first-order reaction rate constant. This assumption can be justified by considering the initial concentration of the excess reactant (oxazole) to be 1 *μ*M. As a result, the *k*_*rxn*_ results from Eq. 3:

3 $$k={k}_{rxn}\times {C}_{Oxazole}\Rightarrow {k}_{rxn}=\frac{k}{{C}_{Oxazole}}=\frac{0.94\,{s}^{-1}}{1\,{\mu M}}=1.56\times {10}^{-15}\frac{c{m}^{3}}{molecule.\,s}$$

**Figure 13:**
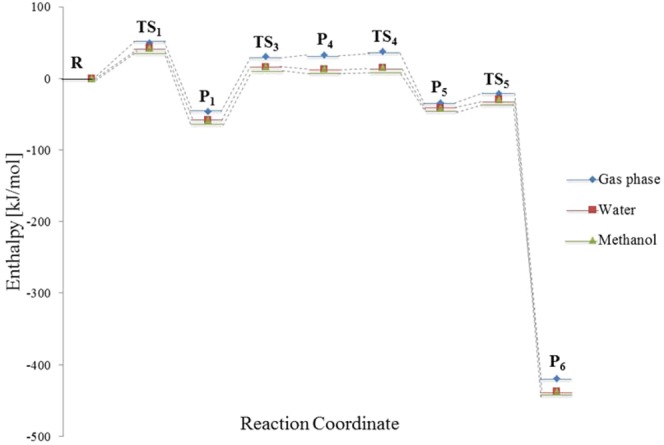
Free enthalpy diagram for main pathway in the mechanism of singlet oxidation of oxazole computed using the PCM solvent model.

Oxazole does not exist in natural form, but as a major segment of important chemicals. As shown in the comparison above, a substituted oxazole (e.g., 2,5-diphenyl-4-methyloxazole) reacts faster with singlet oxygen relatively to an unsubstituted oxazole. The following section treats, in details, the effect of the substituting functional groups on the reaction of oxazole with singlet oxygen. However, for the current leading reaction pathway, the polarisable continuum model (PCM)^33^ accounts for assessing the influence of bulk methanol and water solvents on molecular enthalpies of the reactive agents. As illustrated in Fig. 13, we observed a slight decline in thermal enthalpy, ranging from 7 to 22 kJ/mol as the result of solvent effects.

### Interaction of singlet oxygen with substituted oxazole

#### Electronic effects

This section focuses on the effects of substituent on the reaction pathway of oxazole derivatives with singlet oxygen. Replacing hydrogen atoms with methyl and phenyl functionalities would contribute to molecular electron density variations which could be approached by inductive and resonance electron donating/withdrawing effects. Accordingly, the *σ*-inductive and *π*-resonance effects of methyl and phenyl substituents have been evaluated by means of sEDA and pEDA descriptors, respectively (Table 4). The sEDA descriptor serves as the *σ*-electron donating ability of substituents while pEDA represents their *π*-electron donating potential. The natural population analysis (NPA) was carried out using the Gaussian 09 to attain atomic orbital population symbolised by sEDA descriptor, which has been defined as the sum of valence electrons of *s*, *p*_*x*_, *p*_*y*_ orbitals of all atoms in the oxazole ring. The pEDA on the other hand, is the addition of valence electrons in *p*_*z*_ orbitals.

**Table 4:** Atomic orbital population [e], sEDA and pEDA descriptors for substituted oxazole.


(R1, R2)	*σ*-total [e]	sEDA [e]	(R1,R2)	*π*-total [e]	pEDA [e]
(H, H)	17.51	0.00	(H, H)	5.97	0.00
(Methyl, H)	17.35	−0.16	(Methyl, H)	6.00	0.03
(H, Phenyl)	17.31	−0.20	(H, Phenyl)	5.97	0.00
(Methyl, Phenyl)	17.15	−0.36	(Methyl, Phenyl)	5.98	0.01

**Figure 14:**
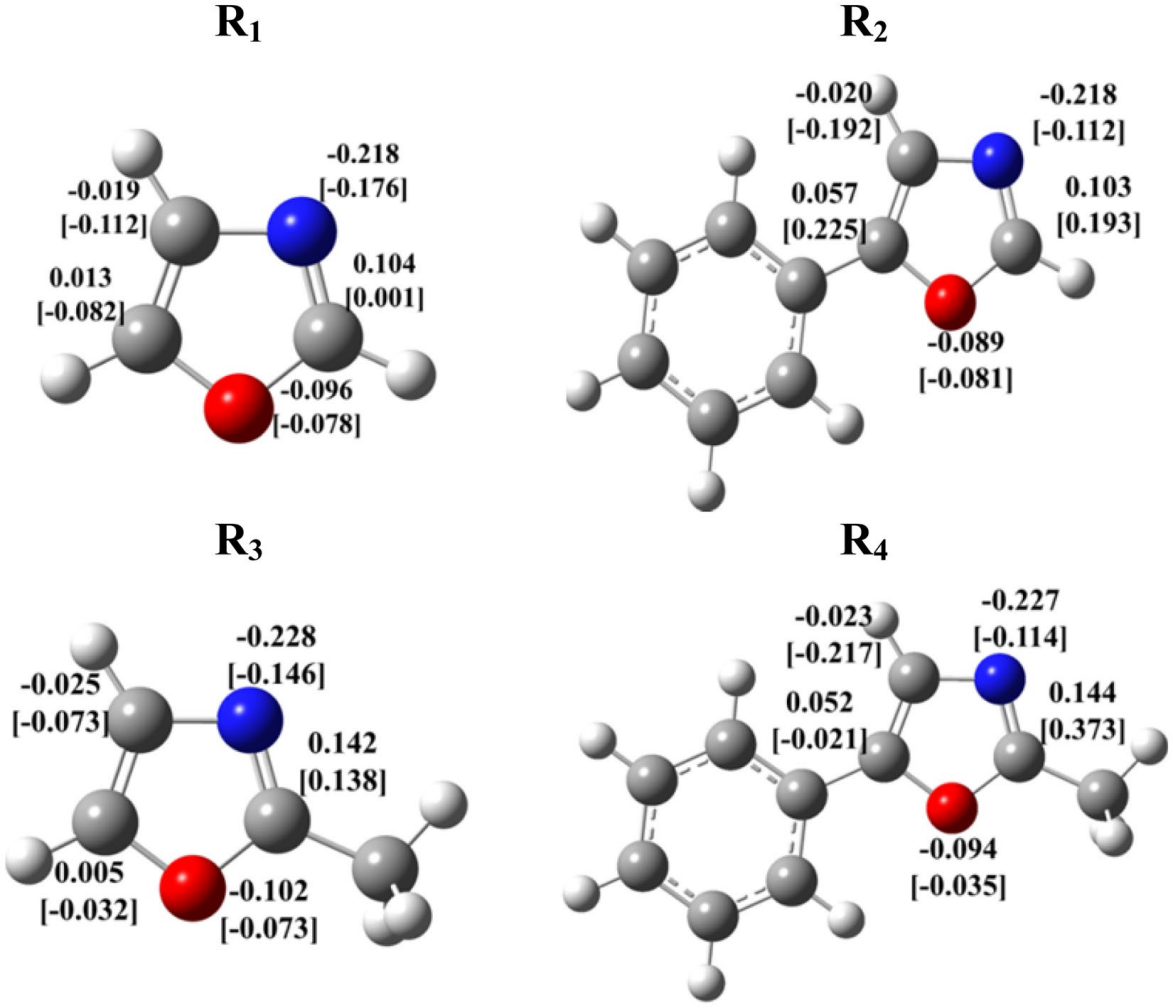
Hirshfeld and Mulliken (in square bracket) electronic charges [e] for in-ring atoms of oxazole derivatives.

As is shown in Table 4, introducing methyl and phenyl functional groups has no notable impact on the *π*-electron distribution on the oxazole ring. However, both substituents reduce the *σ* occupancy of in-ring atomic orbitals, especially phenyl that imposes a stronger electron withdrawing ability via inductive effects. Moreover, we implement the Hirshfeld and Mulliken population analysis to predict the ionic character and partial electronic charges within molecular structures (Fig. 14). Hirshfeld technique is genuinely more accurate approach and less sensitive towards the chosen basis set as compared to the Mulliken^34^ method that suffers a severe dependence on the deployed basis set.

The methyl group induces a growth in the positive Hirshfeld charge on the adjacent ring carbon from 0.104 in parent oxazole (R_1_) to 0.142 and 0.144 in 2-methyloxazole (R_3_) and 2-methyl-5-phenyloxazole (R_4_), respectively. Similar sequence holds true for phenyl functional group, since the positive Hirshfeld charges on the alpha carbons rise from 0.013 in unsubstituted oxazole to 0.057 in R_2_ and 0.052 in R_4_. Therefore, one can conclude that the effective positive charge on the alpha carbon of semi aromatic oxazole ring increases once hydrogen atom is substituted by phenyl and methyl groups, perhaps due to the significant *σ*-electron withdrawal ability of these functionalities, contrary to their feeble *π*-electron donor potential.

#### HOMO-LUMO analysis

**Figure 15:**
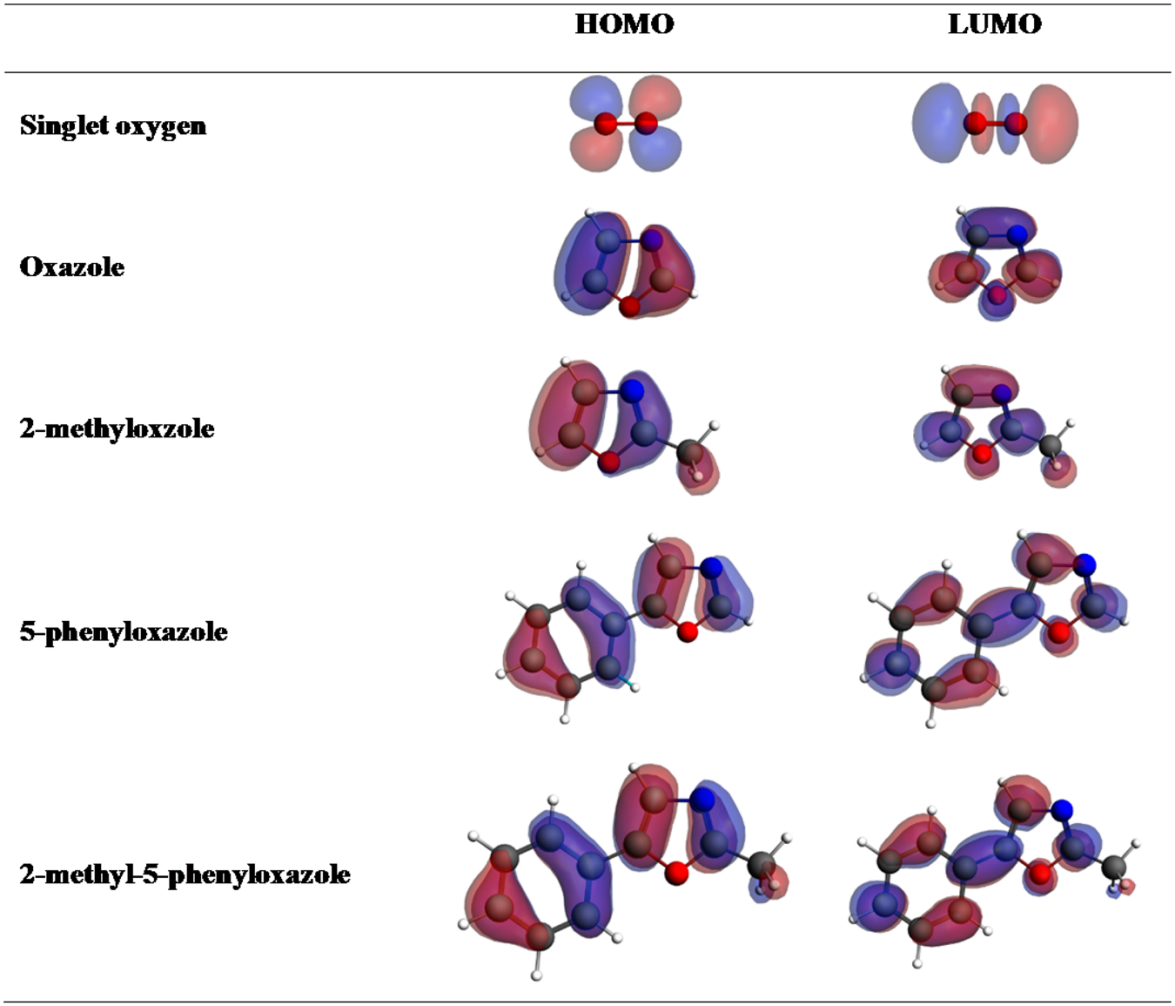
Frontier molecular orbital maps for oxazole and its derivatives (The positive phase is in blue and negative phase is in red).

Figure 15 illustrates the HOMO and LUMO surface contours for singlet oxygen and oxazoles. A chemical reaction will involve the interaction of the HOMO levels of oxazole (electron donors) with LUMO of singlet oxygen (electron acceptor). According to the molecular orbital maps, the methyl functional group barely affects the distribution of HOMO and LUMO contours in oxazole rings. On the other hand, phenyl group slightly pull the electronic densities of LUMO, while the HOMO densities remain intact by this group.

#### Global reactivity descriptors

Table 5 lists the computed energies of frontier molecular orbitals (HOMO and LUMO), energy gap (Δ*E* = *E*_HOMO-_*E*_LUMO_), electronegativity (*χ*), chemical potential (*μ*), global chemical hardness (*η*), global chemical softness (*S*), and global electrophilicity index (*ω*) of selected oxazole moieties.

**Table 5:** Calculated *E*_HOMO_, *E*_LUMO_, *E*_LUMO – HOMO_, electronegativity (χ), chemical potential (μ), global hardness (η), global softness (*S*), electrophilicity index (ω) for oxazole derivatives.

Compound	*E*_HOMO_ [eV]	*E*_LUMO_ [eV]	Δ*E*_LUMO-HOMO_ [eV]	*χ*	*μ*	*η*	*S*	*ω*
Singlet oxygen	−7.271	−5.345	1.926	6.308	−6.308	0.963	1.038	20.660
Oxazole	−7.258	−0.618	6.640	3.938	−3.938	3.320	0.301	2.336
2-methyloxazole	−6.868	−0.337	6.531	3.603	−3.603	3.266	0.306	1.987
5-phenyloxazole	−6.345	−1.542	4.803	3.944	−3.944	2.402	0.416	3.238
2-methyl-5-phenyloxazole	−6.109	−1.401	4.708	3.755	−3.755	2.354	0.425	2.995

The magnitude of the values in Table 5 can assist in analysing the chemical reactivity of the selected compounds with singlet oxygen. A larger gap between HOMO and LUMO levels signifies that the unsubstituted oxazole is a hard molecule with a more stable conformation, which will adversely influence its chemical reactivity. Furthermore, the chemical potential (*μ*) of oxazole derivatives are similarly larger in reference to the singlet oxygen value, justifying their tendency to donate electrons, since electrons flow from a molecule with higher *μ* (lower electronegativity *χ*) to that of lower *μ* (higher electronegativity *χ*). Consistently, the HOMO and LUMO gap, as a measure the electronic excitation from ground state to transition state of molecular structures, in 2-methyl-5-phenyloxazole is the smallest among the selected derivatives of oxazole, suggesting this molecule to be the most reactive.

#### Kinetics and mechanistic investigation

**Figure 16:**
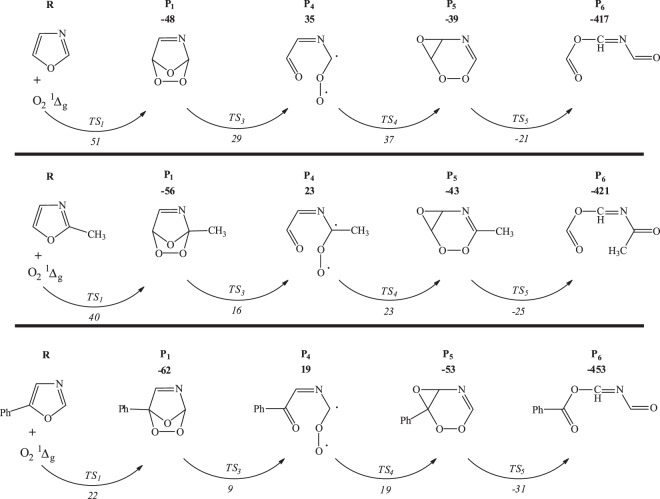
Mechanism of the concerted Diels-Alder addition of singlet oxygen to substituted oxazoles at B3LYP/6–311 ++ g(2df,2p) level of theory. The reported enthalpy values are in kJ/mol.

The effect of introduction of methyl and phenyl functional groups, in the formation of Diels-Alder oxygen-bridged adducts and the subsequent ring cleavage products, is investigated herein. As it is formerly verified in Fig. 7, this type of cycloaddition (i.e., [4 + 2]-) is the main channel through auto-oxidation of oxazole. Figure 16 contrasts the reaction channels and enthalpy barriers involved in oxidative reaction of oxazole derivatives with singlet oxygen. It is clear in Fig. 16 that methyl group boosts the stability of the ring-opening products with respect to the parent oxazole, while the phenyl functionality further enlarges this effect. This fact could mainly be ascribed to the weaker *σ*-electron withdrawal ability of methyl group, as compared to phenyl substituent. The reaction kinetics for the gas-phase photo-oxidation of 2-methyl-5-phenyloxazole and 2,5-diphenyl-4-methyloxazole has been scrutinised to elucidate the energetically favourable pathway.

**Figure 17:**
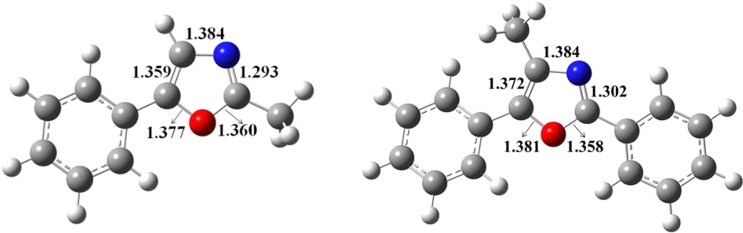
Optimised structures of 2-methyl-5-phenyloxazole (left) and 2,5-diphenyl-4-methyloxazole (right) at DFT/B3LYP method (bond lengths are in Å).

The molecular geometry of these chemicals are optimised at the same level of theory and displayed in Fig. 17. Evidently, the molecules are coplanar and benzene and oxazole rings are aligned in the same plane.

**Figure 18:**
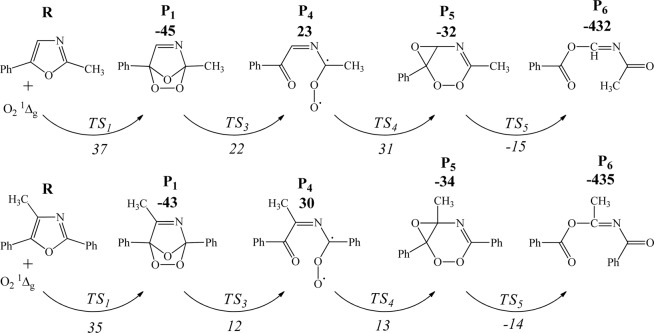
Mechanism of the concerted Diels-Alder addition of singlet oxygen to 2-methyl-5-phenyloxazole [top] and 4-methyl-2,5-diphenyloxazole [bottom] at 6–311 ++ g(2df,2p) and 6–311 g(d,p) basis sets, respectively.

We could not attain convergence for the stabilised transition states and vibrational frequencies of 2,5-diphenyl-4-methyloxazole by applying B3LYP functional combined with highly accurate 6–311 ++ g(2df,2p) basis set. As a result, the smaller 6–311 g(d,p) basis set was selected due to the utility of such basis set in determining the vibrational properties was previously certified encompassing similar root-mean-square (RMS) error in comparison with the larger 6–311 + g(d,p) set^35^. The concerted mechanism of Diels-Alder reactions of substituted oxazole with singlet oxygen is illustrated in Fig. 18.

**Figure 19:**
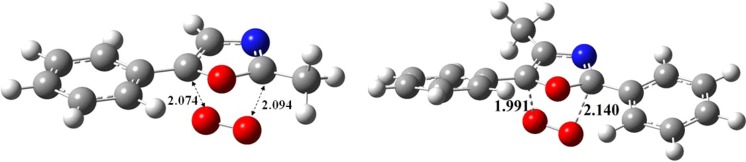
Transition states acquired via DFT/B3LYP method at 6–311 ++ g(2df,2p) and 6–311 g(d,p) basis sets.

According to the obtained computational results, the Diels-Alder interaction of the electron poor singlet oxygen molecule with oxazole derivatives occurs via a low-lying transition state (TS_1_) to synthesise 6-membered endoperoxides (P_1_). Transformation from P_1_ to P_5_ does not proceed through a direct pericyclic reaction. However, the simultaneous cleavage of O-O and C-O bonds in P_1_ structure is attributed to the formation of a highly unstable biradical (P_4_) in which an essentially barrier-less intramolecular rotation provokes ring closure via TS_4_. The cleavage of O-O and C-C bonds in P_5_ constitutes a slight barrier of TS_5_ (17 and 20 kJ/mol) and produces P_6_ via highly exothermic channels. Figure 19 shows the optimised transition states derived from Diels-Alder reaction between singlet oxygen and oxazoles.

Wasserman’s *et al*.^32^ reaction rate constant of dye-sensitised photo-oxidation of 2,5-diphenyl-4-methyloxazole in a number of solvents ranges from 1.61 × 10^7^ M^−1^ s^−1^ (i.e., 67 × 10^−14^ cm^3^ molecule^−1^ s^−1^) in dioxane to 3.92 × 10^7^ M^−1^ s^−1^ (i.e., 6.51 × 10^−14^ cm^3^ molecule^−1^ s^−1^) in benzene. Table 6 assembles the calculated high-pressure limit rate parameters for aryl and alkyl substituted oxazole in gaseous state. As earlier hinted in Section 4.1.2, the substituting functional groups decrease the activation energy, hence faster reaction rates.

**Table 6:** Comparison of kinetic parameters of oxazole and 2-methyl-5-phenyloxazole oxidation by O_2_ (^1^Δ_g_). A is in s^−1^ or cm^3^ molecule^−1^ s^−1^ and *E*_a_ is in kJ mol^−1^.

			
Reaction	*A*	*E*_a_	*A*	*E*_a_	*A*	*E*_a_
Reactant + O_2_^1^Δ_g_ → P_1_	1.68 × 10^−13^	58	1.03 × 10^−13^	44	6.66 × 10^−14^	42
P_1_ → P_4_	7.99 × 10^13^	80	7.85 × 10^13^	70	1.96 × 10^13^	76
P_4_ → P_5_	2.44 × 10^11^	4	3.40 × 10^11^	10	2.19 × 10^11^	2
P_5_ → P_6_	2.17 × 10^13^	21	3.28 × 10^13^	20	1.27 × 10^13^	13

It should be noted that even though the enthalpy of P_4_ (as indicated in Fig. 18) is higher than the transition state (TS_3_), transition states are hypothetical structures corresponding to the energetic state which joins reactants and products. In order to enable the comparison of theoretical and experimental data, we have here assumed that the enthalpy of related transition state (TS_3_) is equal to that of the product (P_4_), and subsequently measured the Arrhenius parameters by ChemRate code taking this assumption into account. The corresponding value of the rate determining step in Table 6 indicates P_1_ → P_4_ conversion at 300 K equals to 1.14 s^−1^ following the first order equation of Arrhenius theory (Eq. 3).

3 $$k=1.96\times {10}^{13}\exp (\frac{-76000}{8.314\times 300})=1.14\,{s}^{-1}$$

Accordingly, a pseudo-first-order reaction rate constant assumes the initial (excess) concentration of 4-methyl-2,5-diphenyloxazole to be 1 μM (a unity concentration for P1), transforming the rate function as follows:

4 $$k={k}_{rxn}\times {C}_{methyl-diphenyloxazole}\Rightarrow {k}_{rxn}=\frac{1.14\,{s}^{-1}}{1\,{\mu M}}=1.14\times {10}^{6}\,{M}^{-1}\,{s}^{-1}$$

The resulting rate constant for substituted oxazole in gaseous phase (Eq. 4) is one order of magnitude less than the literature value, but in reasonable agreement because we have neglected the solvent effect which is supposed to slightly reduce the enthalpy barrier and increase the overall reaction rate as illustrated in Fig. 13.

## Conclusion

This manuscript characterises the kinetic features of the photo-oxidative reaction of oxazole compounds with singlet oxygen, identifying the influence of the practically occurring substituents. To sum up, phenyl (an electron withdrawing group) leads to a decline in the overall electron population of atoms constituting oxazole ring. However, substitution of a methyl group (an electron donating group in reference to hydrogen) on the *sp*^2^ carbon slightly increases the likelihood of an electrophilic attack on oxazole heterocyclic ring by lowering the activation barrier and enhancing the exothermicity of the reaction. The obtained DFT-based gas-phase reaction rates for substituted oxazole conforms to the analogous literature data, justifying the validity of the employed theoretical method adopted in this work. Moreover, polar solvents such as water and methanol reduce the activation energy of the singlet oxygenation of oxazole and subsequently play a noticeable role in increasing the oxidation rate constant.
